# Physiological Candles

**Published:** 1889-10-19

**Authors:** 


### The Doctor's Pulpit.

### PHYSIOLOGICAL CANDLES.

### Compromise.

Candles are getting somewhat out of date in the higher circles of literature and science; and electric lights, or at least fishtail gas-burners, are the only kinds of illumination that are held to be worthy of serious regard. But it must not be forgotten that the "higher circles" of science and literature consist of an exceedingly select few, a rare kind of cream only produced by the purest breeds of literary and scientific cattle; whilst the world, as a world, is composed mainly of "unwashed illiterates" who could not define "bios" or "protoplasm" for their lives. Permit us then, we pray you, the use of a few small candles in order that those who dwell in darkness and in the shadow of physiological death may at least understand that they are in the black night, and that a little light is available for their illumination and help.

But what is a "physiological" candle? The man who should venture on a definition would be an exceedingly rash definer, as rash as he who should attempt to define "bios," and as futile. What a physiological candle may be to others the writer does not know, nor does he care. What he means by it himself he knows very well, and he hopes to enable his readers to discover in due time.

"Great is Diana of the Ephesians," cried the craftsmen of Ephesus when the preaching of a revolutionary doctrine threatened to put an end to their profits, and in a similar strain may it be said, "Great is *compromise* of the English." Not of the "British," be it observed, for the Welsh, the Irish, and the Scotch, but especially the latter, do not find such unlimited virtue in compromise as we do. Your true Scotchman, being a born metaphysician, knows well the difference between "the absolute" and "the conditioned," "the finite" and "the infinite"; or at least he knows that there is a well-understood difference in metaphysics, whatever there may be in the dark and dismal regions where Scottish philosophy is unknown. "Compromise" is eminently an English peculiarity. It is a natural product of the Englishman's mental and moral soil, which soil is poor in comprehension of the absolute, and rich in apprehension of the value of *laissez faire*. The average Englishman cannot comprehend that a thing may be absolutely right or absolutely wrong; that in a given lawsuit one party may be altogether virtuous and the other unmitigatedly vicious; that in a case of slander the slandered may be as innocent as the child unborn, whilst the slanderer may be black as the pit the name of which a polite pen does not care to write; that a man who is at work every day and seems to have little the matter with him, may, notwithstanding, be so desperately ill that he must leave off at once for a sufficient length of time, or go on at the imminent risk of his life. The Englishman, we say, is not the kind of critical animal who sees these things. He discounts the absolutes and pays premiums on the possibles, and thinks himself a "fellow of infinite common-sense." And sometimes, nay very often, this persistent love of compromise and the middle course leads him to conclusions which are not only safe, but, on the whole, just and right.

But in the domain of science, and of physiological as well as other science, compromise is by no means the mighty and magisterial spirit she is in English common life. Science says "Yes" or "No" with the most absolute and uncompromising precision. Your stomach is healthy or it is not healthy; your liver is efficient or it is not; your brain is at its best or it is below par; your heart is perfect and complete in its structural and functional conditions or it is imperfect and unsatisfactory. These are all absolutes. There is positively no place for compromise here. The idea of the patient and his friends, that it may be as the doctor says, but that it does not matter, is entirely opposed to facts, therefore entirely unscientific, and equally, therefore, it is entirely irrational, as irrational as the conduct of the child who puts his finger into the flame of the candle because he is not quite certain that his mother is right when she tells him it is dangerous, and will burn if he touch.

Of course an Englishman, being an Englishman, has a perfect right to pooh-pooh physiology exactly as he has a right to call a foreigner a fool, or to drink brandy and water every day on the West Coast of Africa. By all means let him do as he pleases since, in his own eyes at any rate, even Almighty God will think twice before he speaks roughly to a man of his quality. But he will pay the reckoning some day: he himself, and not his father, or his friend, or his enemy. That is a doctrine which admits of no dispute. In physiology it is altogether impossible to "compound for sins you are inclined to, by damming those you have no mind to." No doubt the physiological energy and robustness of one man is ten times greater than that of another, exactly as one man may be ten or a hundred times richer than another. And just as the richer man may go on for years in wilful extravagance without showing many signs of financial breaking up, so may the man whose stored physiological wealth is great hold on for an almost indefinite length of time without appearing to be in the least injured by his folly. But where is the "plunger" who can be financially reckless for ever? He does not exist; he never did, and he never will exist. The parallel is exact. The man who persistently plays with and misuses his physiological wealth is as certain to reach a crisis and a crash as the dome of St. Paul's would be if a cart load of dynamite were exploded beneath its foundations. The point to be insisted on is the certainty of the thing. No blinding of a man's own eyes; no stopping of his ears; no pouring of contempt upon the doctor; no living of a "jolly life" will either avert the catastrophe or delay it for an hour. No compromise will serve the purpose. The present way of life leads but to one end, and that end can only be averted by one thing, and that is the complete and absolute abandonment of the physiologically wrong and dangerous path.

An example has been selected in which moral and physiological viciousness are combined; and the object of the selection is to make everyone see clearly that vice can only have one result, and to make them approve that result. But physiological vice is not confined to the morally vicious. That is the pity of it. A man may be a perfect angel in his morals, whilst he is foolish, weak, irresolute, and practically worthless from a physiological point of view. Is that his misfortune or his fault? He thinks it is his misfortune. The writer is sure that, in this country at any rate, it is his fault, at least to a large extent. This is quite easily proved by a reference to the plainest teaching of the New Testament. What is the great principle according to which men are condemned by the Christian faith? "This is the condemnation, that light is come into the world, and men love darkness rather than light." Does it really matter, so far as the morality of the question is concerned, what kind of light it is? Is not the main point this, that the light is such as to be of service to man, and that it is so placed as to be easily available? Whatever the ills that afflict the body of the African, he may be able with truth to say that they are misfortunes. But in this country, educated people cannot say so. Physiological light streams in upon them from all sides, but they do not love it. Many, even of the best of them, regard it with indifference, some even with distrust. What a strange creature is man! How exceedingly limited and narrow, even at his broadest and his best!

Some man will say, "You are adding a new burden to life, a new terror." And, indeed, that is exactly the way in which the doctor and the physiologist are regarded by many. It is a new burden, and to a certain extent a new terror, no doubt. But what then? Is the burden a gain and the terror an advantage? That is the question. When the first Napoleon projected an invasion of England a new burden and a new terror were both imposed upon every man in the country; but was not every man glad, not of the danger, but at least to be informed of it, and to bear his part in preparing to resist the invader. The physiologist and the doctor of modern times do not resist facts or make dangers. They merely bring to light the facts or dangers in the midst of which men have lived ever since they began to come together in society. It is clearly every man's deepest interest to welcome the physiological light which has sprung up within the last half century exactly as he welcomes any other kind of light; to welcome it, to use it to the best advantage, and to be grateful for it.

The physiological candle we have held up on the Englishman's persistent and much-trusted habit of "compromise" shows that in the region of bodily health, at any rate, something more definite is wanted. Compromise here is seldom to be relied upon. It is a source of danger. If a man be ill, or merely below par, it is with him as it is with the house which has a slate off the roof, or a brick out of the corner of the wall. What sort of a builder would he be reckoned who proposed to repair the roof by putting on half a slate, or the wall by building in half a brick? Just as foolish is that doctor who treats his patient by half patching him up, or that patient, which is more frequently the case, who will not give the doctor time or opportunity to put more than half a slate on to the battered roof of his health, or half a brick into the crumbling wall. But our physiological candle gives us a hint that if "compromise" be such a doubtful thing in science, there may well also be very strict limits to its value in common practice. That, in fact, is the case. It would be easy to show how, by pulling down the standard of absolute perfection in religion, in politics, in manufactures, in breeding, in habits, and in health, and by substituting a banner of "laissez faire," and of "the middle course is the safest," and all that kind of thing, that incalculable injury is done to the world in its deepest and most significant interests. But that line of thought readers may follow out for themselves. The general principle that "compromise" has only a limited range of value, and that a standard of absolute perfection must always hold its own in the high places of the mind, is indispensable both in morals and in health.

